# The Gut Microbiome as Therapeutic Target in Central Nervous System Diseases: Implications for Stroke

**DOI:** 10.1007/s13311-016-0475-x

**Published:** 2016-10-06

**Authors:** Katarzyna Winek, Ulrich Dirnagl, Andreas Meisel

**Affiliations:** 1 Department of Experimental Neurology, Charité – Universitätsmedizin Berlin, Berlin, Germany; 2 NeuroCure Clinical Research, Charité – Universitätsmedizin Berlin, Berlin, Germany; 3 Center for Stroke Research Berlin, Charité – Universitätsmedizin Berlin, Berlin, Germany; 4 Department of Neurology, Charité – Universitätsmedizin Berlin, Berlin, Germany; 5 German Center for Neurodegeneration Research (DZNE), partner site Berlin, Berlin, Germany; 6 German Center for Cardiovascular Research (DZHK), Partner Site Berlin, Berlin, Germany

**Keywords:** Gut microbiota, stroke, brain–gut microbiota axis, central nervous system, therapeutic target

## Abstract

Research on commensal microbiota and its contribution to health and disease is a new and very dynamically developing field of biology and medicine. Recent experimental and clinical investigations underscore the importance of gut microbiota in the pathogenesis and course of stroke. Importantly, microbiota may influence the outcome of cerebral ischemia by modulating central nervous system antigen-specific immune responses. In this review we summarize studies linking gut microbiota with physiological function and disorders of the central nervous system. Based on these insights we speculate about targeting the gut microbiome in order to treat stroke.

**Electronic supplementary material:**

The online version of this article (doi:10.1007/s13311-016-0475-x) contains supplementary material, which is available to authorized users.

## Introduction

The way we perceive microorganisms and their role in health and disease has changed substantially over the last few decades. It has long been recognized that human–microbial synergy in the gut promotes digestion and contributes to infections when the host–bacterial interaction is disturbed. Recent discoveries, however, have enabled a deeper understanding of the sophisticated interconnection between commensal microbial populations and the host, demonstrating that microbial communities not only refine host metabolism, but also modulate immunity and even contribute to organ development

The first systematic investigations of intestinal bacteria were carried out in the nineteenth century [1]. When investigating stool samples from healthy individuals Friedrich Escherich cultured and characterized the *Bacterium coli commune*, today known as *Escherichia coli* and “the work horse of molecular biology” [2]. Over the following decades more commensal microorganisms were isolated and characterized. But it is only the more recent implementation of advanced high-throughput genetic profiling that has given us detailed insight into the cosmos of intestinal microorganisms. Importantly, gut bacteria create a broad-ranging, dynamic microbial community that contributes substantially to processes in the host organism and reacts to changes in host physiology. Joshua Lederberg suggested the term “microbiota” to describe this complex microbial population and defined it as “the ecological community of commensal, symbiotic and pathogenic microorganisms that literally share our body space” [3]. The microbiome is the collective genome of these symbiotic microorganisms and has a cumulative number of genes larger than that in the human genome [4]. Commensal microorganisms contribute substantially to host metabolism, providing vitamins, extracting nutritional components, and metabolizing xenobiotics. They fortify the intestinal barrier and provide colonization resistance through passive and active competition with potential pathogens [5]. In recent years, important and previously unanticipated functions of commensal microbiota have been described. The gut microbiota has been shown to contribute to the development of the immune system, and, remarkably, also to the development of the central nervous system (CNS) [6].

Thus, it is not at all a surprising suggestion that gut microbiota might play an important role under pathobiological conditions and in physiological processes. Researchers have identified alterations in the composition of gut microbiota in several diseases, such as inflammatory bowel disease [7], diabetes [8], cancer [9], and disorders of the nervous system, for example pain syndromes [10], Parkinson’s disease (PD) [11], spinal cord injury [12], autism [13], and stroke [14–16]. Interestingly, aging is not only a risk factor of stroke, but also affects the gut microbiome [17]. While gut microbiota of older individuals seems to be relatively stable over time, its composition differs substantially from that of younger people and is very heterogeneous [18]. Compelling evidence comes from experimental and clinical studies, and the number of investigations into the role of commensal microbiota in neurological disorders is growing constantly, putting the microbiome to the spotlight in neuroscience. However, the variety and diversity of the diseases, in which the involvement of microbiota has been postulated is tremendous, and only detailed investigations in the coming years will show where the suggested contribution of commensals is legitimate.

Despite all the uncertainties about the role of gut microbiome, commensal microbiota is regarded as a promising therapeutic target and several research groups, start-ups, and large companies have already commenced work on therapies based on the gut microbiome [19, 20]. However, the links between changes in composition of microbiota—which are often the first observation reported in a particular condition—and the course of disease, have to be confirmed as causing or contributing to this condition. This would be the prerequisite for developing microbiome-targeted therapies. In this review we want to discuss evidence for the involvement of gut microbiome in the pathophysiology of CNS disorders and speculate about therapeutic opportunities for manipulations of gut microbiota in CNS disease, with a focus on stroke.

## Microbiota and the CNS: Evidence for a Link

### Impact of Gut Microbiota on the “Health” of the CNS: Experimental Studies

Most insights into the role of microbiota in CNS development and disease originate from studies in germ-free (GF) animals. GF animals have no contact with any microorganisms at any time in their life and are raised in sterile isolators. Establishing the GF animals has proven that life without microbiota is possible [21]; GF organisms, however, have to be provided with exogenous vitamins, otherwise they suffer from severe complications such as vitamin K deficiency, which leads to improper coagulation [21]. Studies in GF mice have revealed that the GF state leads to alterations in the nervous system (Table 1, for a detailed review see [21]). The enteric nervous system in the absence of microorganisms is not fully developed, and GF mice have an altered intestinal motility [22]. The blood–brain barrier in GF mice is more permeable [23], and microglia from GF mice differ in morphology and function from that of conventionally colonized animals [24]. Myelin component genes in prefrontal cortex of GF animals are upregulated, an observation that correlates with an increased thickness of the myelin sheath in axons of GF mice [25]. Remarkably, GF status influences the behavioral phenotype. Compared with specific pathogen-free (SPF) animals, GF animals have increased motor activity and decreased anxiety accompanied by differentially expressed genes involved in synaptic circuitry [26]. Early colonization of GF animals with conventional microbiota reverses this phenotype [26]. GF mice display an exaggerated stress response due to hyper-responsiveness of the hypothalamus–pituitary–adrenal axis [27], and have impaired memory function [28]. Behavioral phenotypes characteristic for particular strains of rodents (e.g., calm, tranquil nature of BALBc mice) can be transmitted upon transfer of microbiota in experiments using GF and conventionally colonized animals [29]. Neurobehavioral changes attributed to obesity, such as depression and dementia, were transferred with gut microbiome in an experimental mouse model [30]. Several studies identified neurochemical changes in the CNS of GF animals. For example, compared with SPF mice, GF mice have an altered hippocampal expression of brain-derived neurotrophic factor (BDNF), but results regarding BDNF levels in GF animals are contradictory [21, 26, 31]. BDNF is an important neurotrophic factor impacting growth and survival of neurons and affecting synaptic transmission [32]. Additionally, male GF mice have elevated levels of hippocampal serotonin and increased levels of plasma tryptophan (serotonin precursor) [33]. Remarkably, microbiota was shown to regulate serotonin synthesis in the periphery [34], and GF animals have lower serotonin levels in plasma [35].

**Table 1:** Differences in central nervous system functions between germ-free (GF) and conventionally colonized mice

• ↑ BBB permeability [23] • Distinct microglia morphology and function (immature microglia phenotype) [24] • Hypermyelination of PFC axons [25] • Behavioral changes, e.g., ↑ motor activity and ↓ anxiety [26], for review see [21] • ↓ Memory functions [28] • Neurochemical changes, e.g., altered BDNF levels [26, 31], ↑ levels of hippocampal serotonin in male GF mice [33], for review see [21] • Hyper-reactive HPA [27]	

↑ = increased; ↓ = decreased; BBB = blood–brain barrier; PFC = prefrontal cortex; BDNF = brain-derived neurotrophic factor; HPA = hypothalamic–pituitary–adrenal axis

Communication pathways between the CNS and microbiota involve immunological, endocrine, metabolic, and neural pathways [36–38]. A direct neural connection between the gut and the brain is provided by the vagus nerve. Intraduodenal injection of *Lactobacillus johnsonii* caused increased gastral vagal nerve signaling and reduced renal sympathetic nerve activity in rats [39]. Long-term treatment with the probiotic bacterium *Lactobacillus rhamnosus* in conventionally colonized mice induced alterations in γ-aminobutryic acid mRNA expression in specific brain regions and reduced anxiety and depression-related behavior, which was mediated by the vagus nerve [40]. Anxiolytic effects have been ascribed to *Bifidobacterium longum* and were apparently mediated by the vagus nerve in a mouse model of chemically induced colitis [41]. The importance of other communication pathways has been suggested, as, in a mouse model, antibiotic-induced changes in behavior were independent of vagotomy or sympathectomy [29]. Disturbances in either of the brain–gut microbiota axis pathways might contribute to the development or modulate the course of CNS disorders.

### CNS Disorders and Microbiota: Experimental Studies

Links between microbiota and CNS diseases have been mainly investigated in rodent models of CNS disorders (Table 2) [21, 37, 42–44], taking the advantage of GF animals. One of the first reports on the role of gut microorganisms for the development of CNS disease originated from the field of studies in an experimental autoimmune encephalomyelitis (EAE) mouse model of multiple sclerosis (MS). Inflammatory T cell-induced destruction of the myelin sheath is a key mechanism in MS pathobiology. Notably, GF mice develop an attenuated form of EAE or even no disease, compared with conventionally colonized mice [45, 46]. This has been attributed to changes in the balance between proinflammatory T helper 17 and anti-inflammatory T regulatory (Tregs) lymphocytes, which is shifted towards protective Tregs in GF animals. Additionally, it has been shown that dendritic cells from GF mice have a reduced ability to elicit proinflammatory responses [46]. In conventionally colonized hosts, gut microbiota has also been identified as important disease modifier in the EAE model. 2D2 myelin oligodendrocyte glycoprotein-specific T cell transgenic mice are prone to develop EAE after immunization [47]. Interestingly, susceptibility for EAE in tumor necrosis factor receptor 2 knockout mice crossed with 2D2 mice is gender specific and associated with distinct microbiota patterns. Females are more prone to EAE, whereas males, having different microbiome composition, are EAE resistant. Additionally, antibiotic treatment ameliorates disease, suggesting a crucial role of microbiota in the development of EAE [48]. Furthermore, EAE has been linked to the dysfunction in the intestinal barrier [49]. In a very recent report, a connection between dietary tryptophan, metabolized by microbiota to aryl hydrocarbon receptor ligands and severity of EAE was established. Tryptophan reduced CNS inflammation over modulation of astrocyte activity, which was mediated by aryl hydrocarbon receptor [50].

**Table 2:** Summary of experimental and clinical studies on gut microbiota in neurological and neuropsychiatric diseases

Studies		
EAE MS	• Only mild form of EAE or no disease after EAE induction in GF mice, linked with shifts in the Th17/Treg balance and ↓ DC functions [45, 46] • ↑ Relative abundance of species *Bacteroides acidifaciens*, *Bacteroides ovatus*, *Akkermansia muciniphila*, ↑ relative abundance of *Oscillospira*, *Anaeroplasma* and *Sutterella in* male TNFR2-ko 2D2 transgenic mice resistant to EAE compared with disease-susceptible females [48] • PSA from *B. fragilis* ameliorates EAE symptoms when given as therapy or prevention [51]	• ↑ Abundance of genera (*Pseudomonas, Mycoplana, Haemophilus, Blautia*, *Dorea*) in patients with MS compared with healthy controls ↑ Abundance of *Parabacteroides, Adlercreutzia*, and *Prevotella* in controls [73] • ↑ Relative abundance of microorganisms from genera pf *Methanobrevibacter* and *Akkermansia*, ↓ relative abundance of *Butyricimonas* correlating with changes in genes regulating immune response, ↓ abundance of *Collinsella*, *Slackia*, and *Prevotella*, ↑ breath methane concentrations in untreated patients with MS compared with healthy controls; treatment linked with alterations in the microbiome composition [74]
Eating disorders	• Possible role of autoantibodies triggered by bacterial proteins in the pathogenesis [54]	• Microbiome of anorexia nervosa patients *vs* healthy controls: ↓ α-diversity, ↑ Bacilli class, unspecified genus in Coriobacteriales family ↓ Clostridia class, order Clostridiales, genera *Anaerostipes* and *Faecalibacterium*; changes in microbiota with weight restoration [136]
Depression	• Different microbiota composition in mice after bilateral olfactory bulbectomy (experimental model of depression) as compared with sham-operated mice [55] • Microbiota essential for the characteristic behavioral phenotype after maternal separation in the mouse model of depression [57]	• ↑ α-diversity and alterations in several bacterial groups of gut microbiota in patients with active major depressive disorder compared with healthy controls, e.g., ↑ relative abundance of genera *Alistipes*, *Blautia*, *Clostridium XIX*, *Lachnospiracea incertae sedis*, *Megamonas*, *Parabacteroides*, *Parasutterella*, *Phascolarctobacterium*, *Oscillibacter*, and *Roseburia* in patients; ↑ abundance of genera *Bacteroides*, *Dialister*, *Faecalibacterium*, *Prevotella*, and *Ruminococcus* in healthy controls [111]
AD	• ↓ Amyloid β pathology in GF AD mice [58] • Antibiotic-induced dysbiosis linked with ↓ amyloid pathology and ↓ gliosis in AD mouse model [59]	
Stroke	• Different microbiota composition in stroke mice compared with sham-operated and naïve animals, ↑ relative abundance of Peptococcaeae, and ↓ relative abundance of Prevotellaceae correlating with lesion severity [15] • ↓ α-diversity with several genera altered, ↓ intestinal motility after stroke; proinflammatory immune cells infiltrating the brain originate from the intestine; fecal transplant with balanced microbiota has neuroprotective effects [16] • ↓ Lesion volume via downregulation of IL-17 γδT cells in mice with dysbiotic microbiota [64] • ↑ Mortality after stroke after extensive depletion of microbiota by antibiotic pretreatment [67] • Worse MCAo long-term outcome after microbiota transplantation from an aged host [60] • ↓ Neuronal injury and ↑ cognitive performance after *Clostridium butyricum* treatment in bilateral common carotid occlusion in diabetic mice (ischemia/reperfusion-induced brain injury) [70]	• Dysbiotic microbiome in patients with stroke and TIA: ↑ genera *Enterobacter*, *Megasphaera*, *Oscillibacter*, and *Desulfovibrio*; ↓ genera *Bacteroides*, *Prevotella*, and *Faecalibacterium* correlating with disease severity. ↓ TMAO in stroke/TIA group compared with patients with asymptomatic atherosclerosis [14] • ↓ Proportion of *Roseburia*, *Bacteroides*, and *Faecalibacterium prausnitzii* in acute stroke compared with healthy controls and patients with irritable bowel syndrome, ↑proportion﻿ of Enterobacteriaceae, Bifidobacteriaceae, and *Clostridium difficile* compared with healthy controls; specific temporal changes of microbial make-up after stroke with restoration after a few weeks [75] • Different microbiota composition in patients with symptomatic atherosclerosis (minor brain infarction = no severe functional deficits, TIA, occlusion of the retinal artery) compared with healthy controls: ↑ *Collinsella* in atherosclerosis patients, whereas ↑ *Roseburia*, *Eubacterium*, and 3 species of *Bacteroides* in controls [81] • No differences in the composition of gut microbiome between symptomatic atherosclerosis patients (minor ischemic stroke, TIA, amaurosis fugax) and controls; several bacterial groups originating possibly from the gut microbiota detected in the atherosclerotic plaques; correlation between microbiome composition and lipid blood profile [78]
TBI	• Correlation of TBI severity with changes in Bacteroidetes, Bacteroidetes family, Porphyromonadaceae, Firmicutes, and Proteobacteria [15]	
SCI		• Differences in gut microbiota composition in patients with SCI compared with healthy controls: ↓ total counts of bacteria from genera *Pseudobutyrivibrio*, *Dialister*, and *Megamonas* in patients with UMN bowel syndrome; ↓ total counts of microorganisms from genera *Roseburia*, *Pseudobutyrivibrio*, and *Megamonas* in patients with LMN bowel syndrome; UMN *vs* LMN ↓ *Marvinbryantia* [12]
PD		• ↓ Abundance of Prevotellaceae in patients with PD compared with controls; specific bacterial groups correlating with motor phenotype [11]
GBS		• Involvement of *Campylobacter jejuni* in the pathogenesis postulated [43]
ASD	• Altered microbiota composition in the mouse model of ASD, most important changes in classes Clostridia and Bacteroidia [53]	• Several studies reporting microbiota changes in children with ASD without consistent results, reviewed in [137]

EAE = experimental autoimmune encephalomyelitis; MS = multiple sclerosis; GF = germ-free; Th = T helper; Treg = T regulatory; DC = dendritic cell; TNFR2-ko = tumor necrosis factor receptor 2 knockout; PSA = polysaccharide; AD = Alzheimer’s disease; IL = interleukin; MCAo = middle cerebral artery occlusion; TIA = transient ischemic attack; TMAO = trimethylamine N-oxide; TBI = traumatic brain injury; SCI = spinal cord injury; UMN = upper motor neuron; LMN = lower motor neuron; PD = Parkinson’s disease; GBS = Guillain–Barré syndrome; ASD = autism spectrum disorder

Interestingly, the therapeutic potential of the microbiota does not appear to depend on the whole microbiota or on specific subpopulations, but rather on several or even single bacterial molecule. For example, the capsular polysaccharide from *Bacteroides fragilis* protects mice from the development of induced EAE, correlating with a shifted balance in the CD4+ cells populations towards interleukin (IL)-10-producing CD4+ FoxP3 Treg cells [51]. Moreover, polysaccharide from *B. fragilis* influences the maturation of the immune answer and regulates the balance between Th1 and Th2 responses [52].

Beneficial effects of *B. fragilis* have also been investigated in the murine maternal immune activation (MIA) model. MIA shares common features with autism spectrum disorder in humans. In this model, pregnant mice are injected with a synthetic poly (I:C) double-stranded RNA mimicking viral infection. The offspring of poly I:C-injected mice display stereotypic behavior and deficits in social interaction and communication. They have altered, dysbiotic, composition of microbiota, accompanied by increased intestinal permeability and distinct intestinal cytokine profiles. Treatment of MIA mice with *B. fragilis* helps to rebalance the composition of microbiota, improves integrity of the epithelial barrier, and reverses behavioral abnormalities [53].

In the field of studies on neuropsychiatric diseases, experimental data suggest involvement of the microbiota in the pathogenesis of eating disorders [54]. Furthermore, altered microbiota composition has been reported in the mouse model of depression [55]. In rodent models, early-life stress alters gut microbiota composition [56, 57], and both stress-induced pathophysiological changes in the host and gut microbiota are necessary for the induction of anxiety-like behavior [57]. Evidence from experimental models suggests involvement of gut microbiota in visceral pain syndromes [37].

Microbiota might also contribute to the development of neurodegenerative CNS disorders, which has been demonstrated in a mouse model of Alzheimer’s disease [58, 59].

### Stroke and Microbiota: Experimental Studies

To date, only a few experimental studies focusing on the role of gut microbiota in cerebral ischemia have been published. Microbiota transferred from an aged host might contribute to deterioration of functional long-term outcome in a mouse model of focal cerebral ischemia [middle cerebral artery occlusion (MCAo)] [60]. However, the exact mechanisms involved in this process are not known [60]. In a rat MCAo model, bacteria translocated from the gut to extraintestinal organs after stroke, which might trigger systemic inflammatory response or even cause poststroke infections [61]. Additionally, stress before stroke might boost bacterial translocation from the intestine into the bloodstream [62]. A recent study demonstrated alterations in microbiota profile after severe cerebral ischemia and linked poststroke dysbiosis with induction of proinflammatory immune response. Transplantation of balanced microbiota after cerebral ischemia improved stroke outcome [16].

In another report antibiotic-induced gut dysbiosis with significantly decreased α-diversity of gut microbiota (diversity within particular habitat [63]) improved outcome and limited the size of ischemic cerebral lesion as measured histologically 3 days after MCAo. These effects were attributed to a decrease in IL-17 producing γδ T cells and an increase in Treg cells in the small intestine, and consequently limited infiltration of harmful IL-17+ γδ T cells to the meninges [64].

Interestingly, similar mechanisms were suggested to explain the effects of high-fat diet on the pathogenesis of type 2 diabetes and obesity. A high-fat diet induced dysbiotic changes in the gut microbiota, impaired function of antigen presenting cells, and decreased number of IL-17 producers, RORγt CD4+ T cells, in the intestine. These alterations preceded the onset of metabolic disease [65]. Obesity and diabetes are, however, well-established risk factors for stroke [66].

Our group investigated the outcome of experimental stroke in C57BL/6 mice after extensive microbiota depletion with broad-spectrum antibiotic pretreatment [67]. We observed significantly increased mortality in microbiota-depleted animals when the antibiotics were stopped before induction of cerebral ischemia. Surprisingly, mortality was linked to acute severe colitis. This phenotype was rescued upon colonization with SPF microbiota or continuous antibiotic treatment. These observations underline the importance of the complex microbial community after cerebral ischemia, when the host immune system is severely compromised by stroke-induced immunodepression [68], which affects immune barriers even in the intestine [69]. We did not observe any effects of microbiota depletion on infarct volume in the brain 1 day after MCAo [67].

Furthermore, model-specific changes in the microbiota after murine MCAo and mild traumatic brain injury were described very recently. In MCAo animals alterations in Peptococcaceae and Prevotellaceace correlated with infarct severity. Additionally, inducing focal cerebral ischemia in mice increased noradrenaline release in the gastrointestinal tract (cecum), and reduced the number of mucoprotein-producing cells and goblet cells. Increased noradrenaline levels and changes in goblet cell function may directly affect the gut microbial community after stroke [15].

A study in diabetic mice identified beneficial effects of supplementation with *Clostridium butyricum* in ischemia/reperfusion-induced brain injury after bilateral common carotid artery occlusion. Treatment with *C. butyricum* decreased neuronal injury and improved the cognitive functions [70]

### Clinical Data

Clinical data on the brain–gut microbiota connection are still scarce (Table 2). Hitherto, clinical microbiota research focused on gastrointestinal, nutritional, or endocrine disorders such as inflammatory bowel disease, obesity, or diabetes type 2.

First clinical reports on the role of gut microbiota indisorders of the nervous system investigated alterations in the composition of the gut microbiome with neurological disease but did not provide a causative link. It was consequently conjectured that gut microbiota plays a role in such different conditions as PD, visceral pain, Guillain–Barré syndrome, stroke, and psychiatric diseases [42, 43, 71]. Changes in the composition of gut microbiota have already been reported in PD. Patients with PD had decreased abundance of Prevotellaceae in their microbiota compared with control subjects. Increased abundance of Enterobacteriaceae was correlated with a more severe motor phenotype [11]. It is further hypothesized that gut microbiota may also play a role in nonmotor symptoms of PD such as neuropsychiatric conditions, sleep disturbances, and pain syndromes [72].

Spinal cord injury is another CNS disorder where the composition of microbiota has recently been investigated. Patients with spinal cord injury have fewer butyrate-producing bacteria in the gut; the consequences of this has not yet been clarified [12]. Moreover, alterations in gut microbiota profiles were observed in patients with MS [73, 74]. Interestingly, immunomodulatory treatment in MS had also effects on the microbiota composition [74].

Changes in the composition of microbiota in the course of stroke were already reported [75], but more importantly, microbiome might contribute to the pathogenesis of the disease, influencing formation of atherosclerotic plaque, as suggested by experimental, as well as clinical data [76–78]. Microbiota is involved in the metabolism of phosphatidylcholine. Levels of its metabolites choline, betaine, and, in particular, trimethylamine N-oxide (TMAO; host product of trimethylamine oxidation), have been identified as predictors of cardiovascular disease risk [76, 77, 79, 80]. TMAO is also produced during the metabolism of L-carnitine, which is found in red meat [80]. All of these metabolites increase cholesterol accumulation in macrophages and promote foam cell formation [77]. Conversely, symptomatic atherosclerosis was linked to changes in the composition of gut microbiome [81].

A recent clinical study compared microbiome composition in patients diagnosed with asymptomatic atherosclerosis, stroke, and transient ischemic attack. The trial data also included patient TMAO plasma levels. Surprisingly, the asymptomatic atherosclerosis group with and without carotid plaques had similar levels of TMAO and comparable gut microbiota composition. In contrast, patients with stroke and transient ischemic attack differed substantially from the asymptomatic subjects regarding microbiome composition, but their TMAO levels were lower than those of the asymptomatic group [14].

In another study, serum levels of carnitine and its metabolite γ-butyrobetaine—rather than TMAO and trimethyllysine—were elevated in patients with carotid atherosclerosis. Serum levels of gamma-butyrobetaine and trimethyllysine were also associated with cardiovascular death [82]. Conflicting evidence on gut microbiota metabolites and atherosclerosis was also submitted by experimental studies. Atherosclerotic plaque formation was increased in apolipoprotein E knockout transgenic mice (ApoE^–/–^; mouse model of atherosclerosis [83]) when the mice were supplemented with L-carnitine [80]. ApoE^–/–^ animals, however, lack an important enzyme cholesteryl ester transfer protein, which transfers cholesterol ester between lipoproteins of high and low density. Interestingly ApoE^–/–^ transgenic mice overexpressing human cholesteryl ester transfer protein supplemented with L-carnitine had decreased aortic atherosclerotic plaque formation and high TMAO levels [84]. Moreover, in contrast to conventionally colonized ApoE^–/–^ mice, the same transgenic animals housed under GF conditions developed severe atherosclerosis when fed a low cholesterol diet [85].

These data suggest that metabolites of the microbiome might also provide a certain level of protection from the development of disease. It should be noted that gut microbiota studies in the clinical settings are more challenging than experimental studies in animals. Study populations in humans are usually more heterogeneous, as they are influenced by multiple factors independent of the disorder under investigation. These confounders might affect the composition of the intestinal microbial community as well [71]. Furthermore, as illustrated by the example of studies in atherosclerosis, microbiota may confer dualistic effects on the host—protective and harmful—in the pathogenesis and course of the disease [86] .

## Open Issues and Future Avenues of Research

### Gut Microbiota as Therapeutic Target in Stroke

**Fig. 1:**
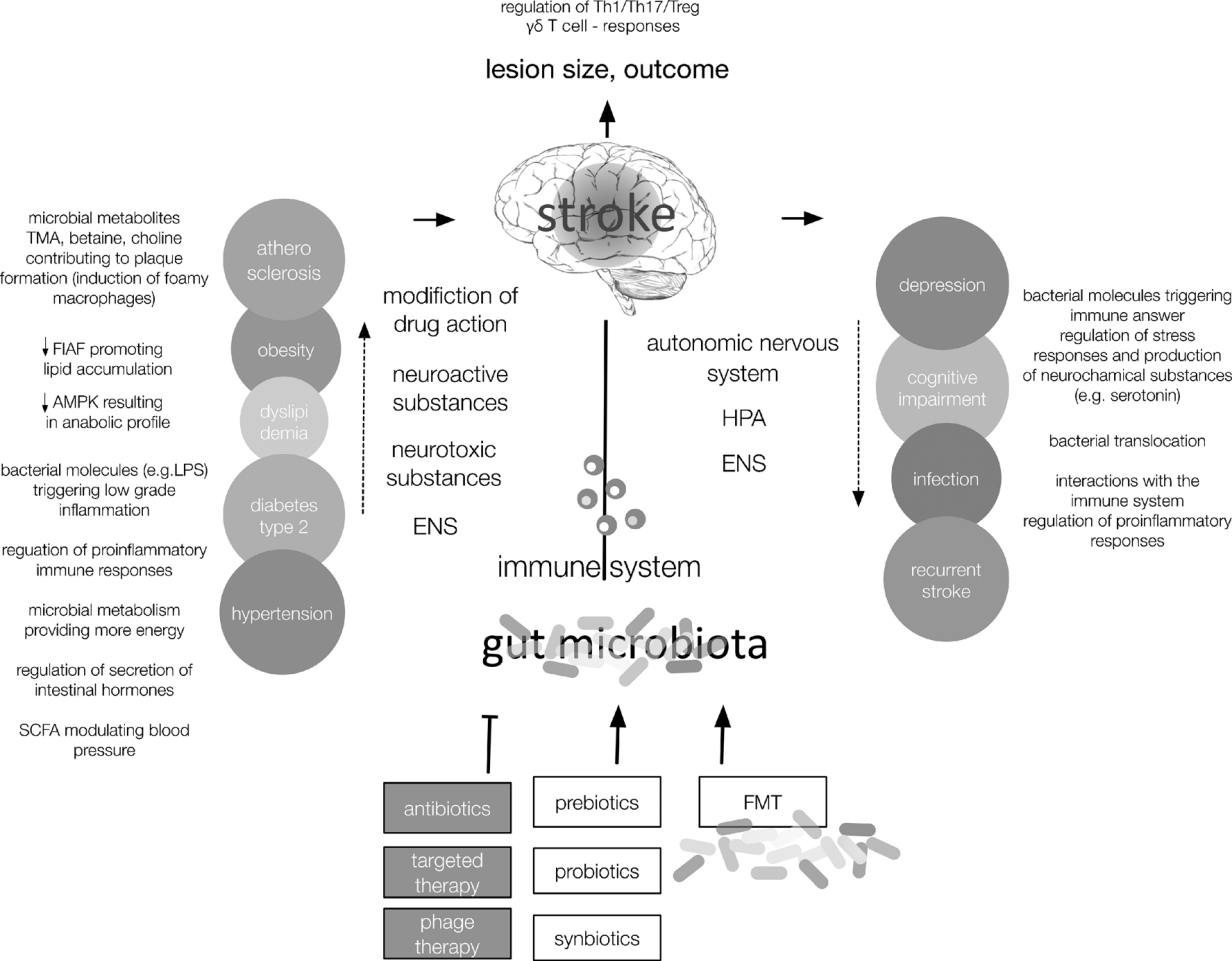
Modifiable stroke risk factors and poststroke complications with hypothesized involvement of the gut microbiota. Gut microbiota might contribute to the pathogenesis of cerebrovascular disorders and serve as a therapeutic target for modulating stroke-related risk factors, neuroinflammatary responses, and complications after stroke. The interplay between the gut microbiome and the host involves the host immune system, hypothalamus–pituitary–adrenal glands axis (HPA), autonomic nervous system, enteric nervous system (ENS), and microbial molecules and metabolites. Th = T helper; Treg = T regulatory; FMT = fecal microbial transplantation; FIAF = fasting-induced adipose factor; AMPK = 5' adenosine monophosphate-activated protein kinase; LPS = lipopolysacharide; TMA = trimethylamine; SCFA = short-chain fatty acids

Gut microbiota could be a potential therapeutic target for diseases that constitute risk factors for stroke and/or for complications after cerebral ischemia (Fig. 1). Experimental and clinical evidence suggests that microbiota in combination with a high-fat diet contributes to the development of type 2 diabetes [87], obesity [65, 87–91], and hypertension [92, 93]. Elevated TMAO plasma levels, linked with adverse cardiovascular events in humans, were normalized by antibiotic treatment [79]. However, prolonged antibiosis for atherosclerosis prevention is potentially harmful as it poses an increased risk of antibiotic resistance and detrimental infections such as *Clostridium difficile* colitis. In a more sophisticated approach, TMAO production has been successfully inhibited by 3,3-dimethyl-1-butanol, a structural analog of choline and an antagonist for the microbial trimethylamine lyase [94].

Further, microbiome-based therapy appears to be a promising approach for the treatment of poststroke complications. Complications after cerebral ischemia comprise, among others, bacterial infections, cognitive impairment, and depression [95, 96]. In particular, stroke-associated pneumonia is considered to contribute to detrimental neurological outcome. We would speculate that these conditions can be targeted via the gut microbiome. Although experimental findings are equivocal, gut microbiota might be the source of systemic infections in patients with stroke, particularly when host immunity and immunological barriers are compromised by poststroke immunodepression [97]. In a mouse model gut microbiota has been demonstrated to support the host immune defense against pneumococcal pneumonia [98]. After microbiota depletion with antibiotic treatment, C57BL/6 mice infected with *Streptococcus pneumoniae* showed higher mortality, more severe organ damage, increased spread of the pathogen, elevated inflammatory markers, and limited alveolar macrophages functions than did untreated mice. Transfer of fecal microbiota from untreated animals reduced bacterial counts in the lungs and normalized inflammatory markers. [98]. Whether the gut microbiota or rather the microbiota of the respiratory tract is responsible for these effects remains a matter of debate. Both microbial communities are affected by the antibiotic treatment used in this study to deplete the microbiota, as well as by fecal transplantation over oral gavage, implemented to restore microbial community [99].

In current practice patients with stroke are often treated with antibiotics in the acute course of the disease due to poststroke infections. Certainly, antibiotic treatment will target not only pathogens in lung and urinary tract, but also the commensal bacterial populations in other organs such as the gut. Antibiotics induce rapid and long-lasting changes in the microbiome [100]. Treatment with clindamycin over 7 days triggered shifts in the microbiota, detected even 2 years after the termination of the antibiotic therapy [101]. Patients with severe strokes are usually treated for 5 to 10 days and often with combinations of broad-spectrum antimicrobial agents [102]. In experimental stroke, profound depletion of microbiota by extensive broad-spectrum antibiotic pretreatment to eliminate cultivatable bacterial microorganisms causes detrimental outcome due to acute and severe colitis [67].

Investigations in an animal model [103], as well as in a clinical study [104], suggest that stroke triggers a metabolic switch to a catabolic profile, causing sarcopenia and substantial weight loss. Although the underlying mechanisms are not fully elucidated, involvement of commensal bacteria is likely. It has been suggested that shifts in microbiota are associated with weight changes [105]. Altered composition of gut microbiota has been observed in a mouse model of acute leukemia accompanied by cachexia [106]. Restoration of specific microbial species led to reduction of the inflammatory cytokines and markers involved in the protein breakdown in skeletal muscle [106].

Stroke survivors often suffer from neuropsychiatric complications, mainly poststroke depression [95]. It has been shown that a multitude of mechanisms, including systemic inflammatory processes, are involved in the manifestation of poststroke depression [107]. However, it is also reasonable to assume a role for gut microbiota, as experimental and clinical studies suggest a causative role of microbiota in mood disorders [108–111].

The abovementioned relations are largely speculative but ultimately testable. They provide examples for how gut microbiome might serve as target to treat or even prevent conditions that pose risk factors for stroke or poststroke complications.

### Therapeutic Manipulation of the Microbiome?

Principally, there are two strategies for manipulating microbiota: 1) target a defined microorganism by direct elimination, modification, or triggering of the immune response via vaccination with a specific epitope; and 2) manipulate whole communities either individually or by combinations of antibiotics, probiotics, prebiotics, synbiotics, or fecal transplant [112].

Targeting specific microbiota is promising when a causal link has been demonstrated between a particular microorganism and a disease. The approach of targeting whole communities appears to be a shot in the dark: the outcome of such a therapy is not only unpredictable, but might also have more severe adverse effects, in particular when the entire microbial community is profoundly influenced. As already discussed, antibiotic treatment might shift the gut microbiome incurring detrimental outcome, in particular in immune compromised patients with stroke. In contrast, several probiotics have been shown to exert beneficial effects on the human immune system [113], while difficulties can and do arise owing to the fact that some bacterial strains are not even able to survive the human upper gastrointestinal tract, and often do not colonize the gut. The choice of which probiotic strain to use as a treatment is therefore complicated. Commonly, probiotic supplementation is considered to be well tolerated. However, treatment with probiotics might have harmful consequences. For example, the PROPATRIA (PRObiotics in PAncreatitits TRIAl) trial had to be stopped prematurely owing to significantly higher mortality in the group treated with probiotics [114, 115]. Moreover, several immunocompromised patients or newborns are reported to have suffered from sepsis due to probiotic microorganisms [116–118]. These findings advise caution, given that stroke induces an immune depression [68].

Ever since 1958, transfer of a whole microbiota community from a healthy donor, so-called fecal microbiota transplantation (FMT), has been established in the treatment of patients with severe *C. difficile* colitis. At that time the causative agent had not even been identified [119]. The effectiveness of FMT in pseudomembranous colitis was recently confirmed by a clinical study comparing FMT with vancomycin treatment [120]. However, the optimal way of delivering the “transplant” to the gut has not yet been established. Studies are being carried out to improve fecal bacteriotherapy in order to recolonize the gut with microorganisms from healthy donors [121]. Delivering fecal microbiota by pill allows standardization and might reduce risks associated with the more invasive delivery [122]. However, standards for identifications of persons who qualify as healthy donors are also not yet fully established. Since the field of microbial therapy is in its infancy and little is known about side effects, transplantation of currently unknown pathogenic microorganisms by FMT cannot be excluded. Some undesirable and hitherto unpredictable effects of FMT have been already reported, for example peripheral neuropathy [123]. Moreover, in the experimental setting, transplantation of intestinal microbiota from an atherosclerosis-prone to a disease-resistant mouse strain was sufficient to transmit susceptibility to atherosclerosis [124]. Thus, even before risk factors for diseases manifest, gut microbiota might have pathogenic potential, making the definition of healthy persons as donors for FMT inherently difficult. Nonetheless, stool transplant has already been approved in USA by the Federal Drug Administration for treating *C. difficile* enteritis that is nonresponsive to standard therapies; however, the legal status of FMT is still being debated [125]. Furthermore, novel therapeutic approaches, specifically targeting microbiome are developing rapidly, including phage therapy, targeting quorum-sensing molecules, or certain bacterial genes [100, 126].

High expectations for developing new microbiota-based therapies are pinned on the microbial interplay with the immune system and the metabolic capacity of the microbiome. Gut microbiota possesses metabolic capacities comparable with those of a human liver [127]. Products of bacterial fermentation, short-chain fatty acids, modulate immune responses [128], and might contribute to the pathogenesis of hypertension [93]. Moreover, gut bacteria can directly metabolize drugs to influence their activity or indirectly modify host responses to xenobiotics [129]. This has implications for the bioavailability of drugs and their toxicity. For example, levodopa, used in the treatment of PD, needs to be decarboxylated to dopamine in the CNS. Intestinal bacteria are also capable of decarboxylating the drug [130]. However, levodopa, but not dopamine, is able to cross the blood–brain barrier, so decarboxylation outside the CNS would affect its brain availability [131].

In an experimental mouse study, nonlethal inhibition of bacterial β-glucuronidases, which reactivate the colon cancer drug CPT-11 (Irinotecan), lead to alleviation of the drug's toxicity [132]. These effects seem to depend on a specific composition of gut bacteria. Gut microbiota (and, specifically, *Bacteroides* species) has proved to be indispensable for efficacy of certain anticancer drugs (Ipilimumab, anti-cytotoxic T-lymphocyte-associated protein 4), which are unsuccessful in GF or antibiotic-treated mice [133]. *Bifidobacterium* promoted success of antitumor therapy with anti-programmed cell-death-ligand 1 by boosting T-cell responses [134].

Taken together, composition of gut microbiota might influence treatment success of certain drugs through its microbial metabolic functions or by modulating the immune response. This field of research certainly needs to be followed up to disentangle the therapeutic potential of the commensal microorganisms and help to predict individual responses to certain therapies. It appears very likely that many commonly used drugs, not investigated in the context of gut microbial composition, might exert their effects, at least in part, on microorganisms of the gut.

One of the main obstacles in current microbiome-based treatment research is the definition of measurable and clinically relevant microbiota-related endpoints for predicting the success of the therapy [135]. There is no generally accepted method or even gold standard for monitoring microbiota that could be implemented in the clinical setting. More importantly, relevant endpoints for efficacy, comparable with clinical trials investigating drugs for treatment, need to be used to prove a causative link between a certain microbiota composition and a particular condition, and to provide a novel microbiome-based treatment strategy [135].

In summary, gut microbiota research is a flourishing field with novel and often surprising discoveries about the role of commensal microorganisms in health and disease. Thanks to the advances in sampling and sequencing techniques, the detailed characterization and profiling of microbiome is now feasible. Although the concept of gut microbiota–brain communication seems to be established now, investigations are still needed to characterize the mechanistic connections between changes in the gut microbiota and neurological diseases. This would be the prerequisite for developing successful microbiota-based therapies in CNS disorders, the effectiveness and safety of which would need to be tested in experimental studies and large clinical trials. Although most of this research has been done outside neurosciences, we provided examples, which we believe to be instructive for stroke research.

## Electronic supplementary material

Below is the link to the electronic supplementary material.

ESM 1 (PDF 1225 kb)
